# Experimentally induced colitis impacts myelin development and home-cage behavior in young pigs regardless of supplementation with oral gamma-cyclodextrin-encapsulated tributyrin

**DOI:** 10.3389/fnins.2025.1484497

**Published:** 2025-03-31

**Authors:** Loretta T. Sutkus, Kaitlyn M. Sommer, Zimu Li, Bradley P. Sutton, Sharon M. Donovan, Ryan N. Dilger

**Affiliations:** ^1^Neuroscience Program, University of Illinois, Urbana, IL, United States; ^2^Department of Animal Sciences, Division of Nutritional Sciences, University of Illinois, Urbana, IL, United States; ^3^Department of Bioengineering, University of Illinois, Urbana, IL, United States; ^4^Beckman Institute for Advanced Science and Technology, University of Illinois, Urbana, IL, United States; ^5^Department of Food Science and Human Nutrition, University of Illinois, Urbana, IL, United States; ^6^Division of Nutritional Sciences, University of Illinois, Urbana, IL, United States

**Keywords:** brain development, colitis, dextran sodium sulfate, gamma-cyclodextrin encapsulated tributyrin, magnetic resonance imaging

## Abstract

**Introduction:**

Colitis, a chronic intestinal disorder that causes inflammation of the colonic mucosa, has been linked with structural brain abnormalities. To combat intestinal inflammation, researchers have investigated how nutritional supplementation, such as butyric acid, may ameliorate untoward effects. By encapsulating and using conjugates of butyrate, such as butyrate glycerides (i.e., tributyrin), slower release to the lower portions of the gastrointestinal tract can be achieved. Additionally, butyrate supplementation has been linked with supporting brain function and regulating integrity.

**Methods:**

In the present study, a total of 24 intact male pigs were artificially reared and randomly assigned to 1 of 3 treatment conditions: (1) a control milk replacer (CON), (2) control plus oral dextran sodium sulfate (DSS) to induce colitis, or (3) control supplemented with 9.0 mM of gamma-cyclodextrin encapsulated tributyrin (TBCD) plus oral DSS (TBCD+DSS). Pigs were orally administered DSS treatments daily from postnatal day (PND) 14–18. Continuous video recording began on PND 3 and ceased on PND 27 or 28, with videos processed and analyzed for home-cage tracking behavior. On PND 26 or 27, pigs underwent neuroimaging procedures to assess overall brain anatomy (MPRAGE), microstructure (DTI), and myelin (MWF).

**Results and discussion:**

Home-cage spatial preference was not altered prior to DSS dosing or during the overall study period. However, TBCD+DSS pigs spent less (p < 0.05) time within quadrant 4 when compared with CON pigs. Across almost all 29 brain regions assessed, absolute volumes were observed to be smaller in the TBCD+DSS group compared with CON and DSS groups. However, once individual volumes were assessed relative to the whole brain, most treatment effects dissipated other than for gray matter volume (p = 0.041). Diffusivity was found to be altered in several regions across treatment groups, thereby indicating differences in fiber organization. In areas like the hippocampus and thalamus, when fractional anisotropy (FA) values were highest for a given treatment, in the other diffusion metrics (mean, radial, axial diffusivity) values were lowest for that same treatment, indicating more organized cellular structure. Several other diffusion trends and differences were observed across various regions. Lastly, myelin water fraction (MWF) values were lowest in DSS-treated groups compared with CON (p < 0.05) for the whole brain and left/right cortices.

**Conclusion:**

Overall, fiber organization and myelination were observed to be altered by experimentally induced colitis and contrary to expectations, tributyrin supplementation did not ameliorate these effects. Future work is warranted to investigate other protective nutritional mechanisms for colitis.

## 1 Introduction

Colitis is a prevalent chronic digestive disorder that causes inflammation of the colonic mucosa (Langan et al., 2007). Characterized by intermittent bloody diarrhea and abdominal pain, colitis has been associated with various extraintestinal manifestations (Langan et al., 2007; Scheid and Teich, 2007) such as inflammatory demyelinating neuropathies (Lossos et al., 1995). Specifically, colitis has been associated with multiple sclerosis and acute disseminated encephalomyelitis in both human and non-human primates (Scheid and Teich, 2007). Additionally, other studies have reported associations between chronic bowel disorders and psychological comorbidities (Abautret-Daly et al., 2018) and structural brain abnormalities (Kornelsen et al., 2021; Vitali et al., 2022). There is evidence to support the influence that chronic digestive diseases can have on the brain by altering the physiological permeability of several immune and vascular barriers (Carloni and Rescigno, 2022). However, the direct impacts remain ambiguous and limited work has explored how early-life brain development may be impacted. Additionally, although 10% of inflammatory bowel diseases have a pediatric onset, research has mostly focused on growth impairments and future comorbidity risks rather than its influence on brain development (Bouhuys et al., 2023). Given the variable nature of clinical research, using animal models to explore further implications of colitis on development is imperative as they provide a more controlled environment for investigation. To induce colitis in animal models, dextran sodium sulfate (DSS) is an established and commonly used method (Cao et al., 2018; Herías et al., 2005; Lackeyram et al., 2017; Yao et al., 2010), which has been reported to follow similar disease progression as human inflammatory bowel disorders (Oh et al., 2014).

Various studies have explored the ability for nutritional supplementation to influence the effects of colitis (Cao et al., 2018; Herías et al., 2005; Yao et al., 2010) with recent work highlighting administration of butyrate in particular (Venkatraman et al., 2003; Vieira et al., 2012; Xiao et al., 2021). Butyrate metabolism is inhibited by colonocytes in mice induced with experimental colitis (Ahmad et al., 2000) and by supplementing it into the diet, inflammatory responses have been attenuated (Vieira et al., 2012). Supplementation of direct butyrate into the diet presents challenges as it has a strong odor and is rapidly absorbed in the upper gastrointestinal tract (Bedford and Gong, 2018). To combat this, various forms of butyrate, such as tributyrin (a butyrate glyceride), have been encapsulated to minimize unpleasant odor and taste as well as ensure that butyrate is released at the target location of the gastrointestinal tract (Bedford and Gong, 2018; Shi et al., 2020). Furthermore, supplementation with sodium butyrate has been reported to support brain function (Lynch et al., 2021), specifically through regulating gene expression (Alpino et al., 2024) facilitating oligodendrocyte differentiation (Chen et al., 2019), and increasing neurogenesis and cell proliferation in the hippocampus of rodents (Kim et al., 2009; Yoo et al., 2011). Butyrate has also been observed to be able to cross the blood brain barrier and it is believed to regulate its integrity (Alpino et al., 2024). Through these implications, it is of interest to investigate the potential for butyrate to ameliorate colitis as well as its capabilities to alter brain development.

Throughout the scientific literature, rodents have been a commonly utilized model in investigating various extraintestinal manifestations associated with colitis, specifically regarding brain outcomes (Do and Woo, 2018; Gampierakis et al., 2021; Han et al., 2020). Therefore, there is room for a more translatable animal model and utilizing the pig is advantageous due to the many gastrointestinal and neurological features shared with humans (Lind et al., 2007; Miller and Ullrey, 1987). Additionally, the pig has been widely used to investigate how nutritional supplementation can affect brain development using neuroimaging procedures (Golden et al., 2023; Joung et al., 2020; Mudd et al., 2017, 2018; Mudd and Dilger, 2017). Furthermore, pigs have been frequently utilized as animal models for gastrointestinal diseases, such as inflammatory bowel diseases and necrotizing enterocolitis, since their gastrointestinal development and neurodevelopmental trajectories and milestones are similar to humans (Mudd and Dilger, 2017; Yin et al., 2017). Therefore, in the current study, home-cage behavior and noninvasive neuroimaging were acquired in pigs to assess the impacts of colitis and encapsulated tributyrin supplementation on brain development. Multiple neuroimaging modalities were captured to obtain macrostructural, microstructural, and myelin properties of the brain. We hypothesized that supplementation with a conjugate of butyrate would support brain development in the pig, specifically in an experimentally induced colitis model.

## 2 Materials and methods

All described experimental procedures were approved by the University of Illinois Urbana-Champaign Institutional Animal Care and Use Committee as congruent with the Guide for the Care and Use of Laboratory Animals.

### 2.1 Animal care and housing

Twenty-four intact (i.e., non-castrated) male pigs were obtained from a commercial swine herd on postnatal day (PND) 2 and artificially reared in the University of Illinois Piglet Nutrition and Cognition Lab (PNCL) until study conclusion on PND 27 or 28. The study was completed in 4 separate cohorts of pigs to accommodate neuroimaging and collection logistics. To ensure genetic similarity, pigs were obtained from Pig Improvement Company (PIC; Hendersonville, TN) Line 3 dams that were artificially inseminated using a pooled semen source that included between 50 and 150 boars. Prior to experimental randomization by body weight, pigs received both a 5-mL subcutaneous and 3-mL oral dose of *Clostridium perfringens* antitoxin C and D (Colorado Serum Company, Denver, CO). Pigs were individually housed in pig-rearing units, referred to as home-cages, that allowed pigs to see, hear, and smell each other without direct contact. Detailed descriptions of PNCL rearing and housing are reported by Fil et al. (2021).

#### 2.1.1 Experimental conditions

Pigs were randomly assigned to one of three experimental conditions: (1) a nutritionally adequate milk replacer (CON; Purina® Multi-Species Milk Replacer, Purina Animal Nutrition LLC, North Arden Hills, MN, United States), (2) CON diet plus oral administration of DSS (DSS), and (3) CON diet with 8.3 g/kg of gamma-cyclodextrin encapsulated tributyrin (TBCD) plus oral administration of DSS (TBCD + DSS). Pigs received *ad libitum* access to liquid milk replacer treatments starting on PND 3 and continuing until study conclusion.

TBCD was manufactured (Shi et al., 2020) and administered (Bartholome et al., 2004) based on previously reported procedures and recommendations. DSS and TBCD+DSS treatment groups were orally administered DSS at 1.25 g/kg body weight daily from PND 14 to 18. Thirty minutes prior to scheduled milk replacer feeding each morning, pigs in the DSS and TBCD+DSS treatments received their respective dose in their milk bowl for voluntary ingestion. Health checks, feed disappearance, and body weights were obtained daily, and associated descriptions/outcomes have been reported separately (Sommer et al., 2022).

### 2.2 Home-cage tracking

Each home cage was fitted with a ceiling-mounted camera (Lucid Vision Labs; Richmond Canada) that recorded video with a resolution of 1,024 × 1,024 at 20 frames per second. Video data was temporarily stored on individual servers, then integrated storage was achieved through a dedicated video management system (Motif V5; Loopbio, Vienna, Austria). Utilizing this system, pigs were continuously recorded within the home cage from PND 3 to 27 or 28. On PND 27 or 28, camera recording ceased, and videos were analyzed for pig tracking.

Pig location within the home cage was tracked utilizing a deep learning framework (Detectron2) that had been trained to utilize hundreds of images of pigs within their home cage at PNCL. This training was done by manually generating a bounding box around each individual pig. As the model was trained utilizing human-generated bounding boxes, the accuracy of the tracking models was limited to the accuracy of the training model. Once the model was trained, the center of each bounding box was identified to indicate the center of mass for each pig, allowing for predictions of the animal’s approximate location. This process generated (x, y) coordinates for the current position of the pig on a per-frame basis. With the use of the (x, y) coordinates, the UNIX epoch time of each recorded frame was stored to provide an anchor for post-processing relative time.

**Figure 1:**
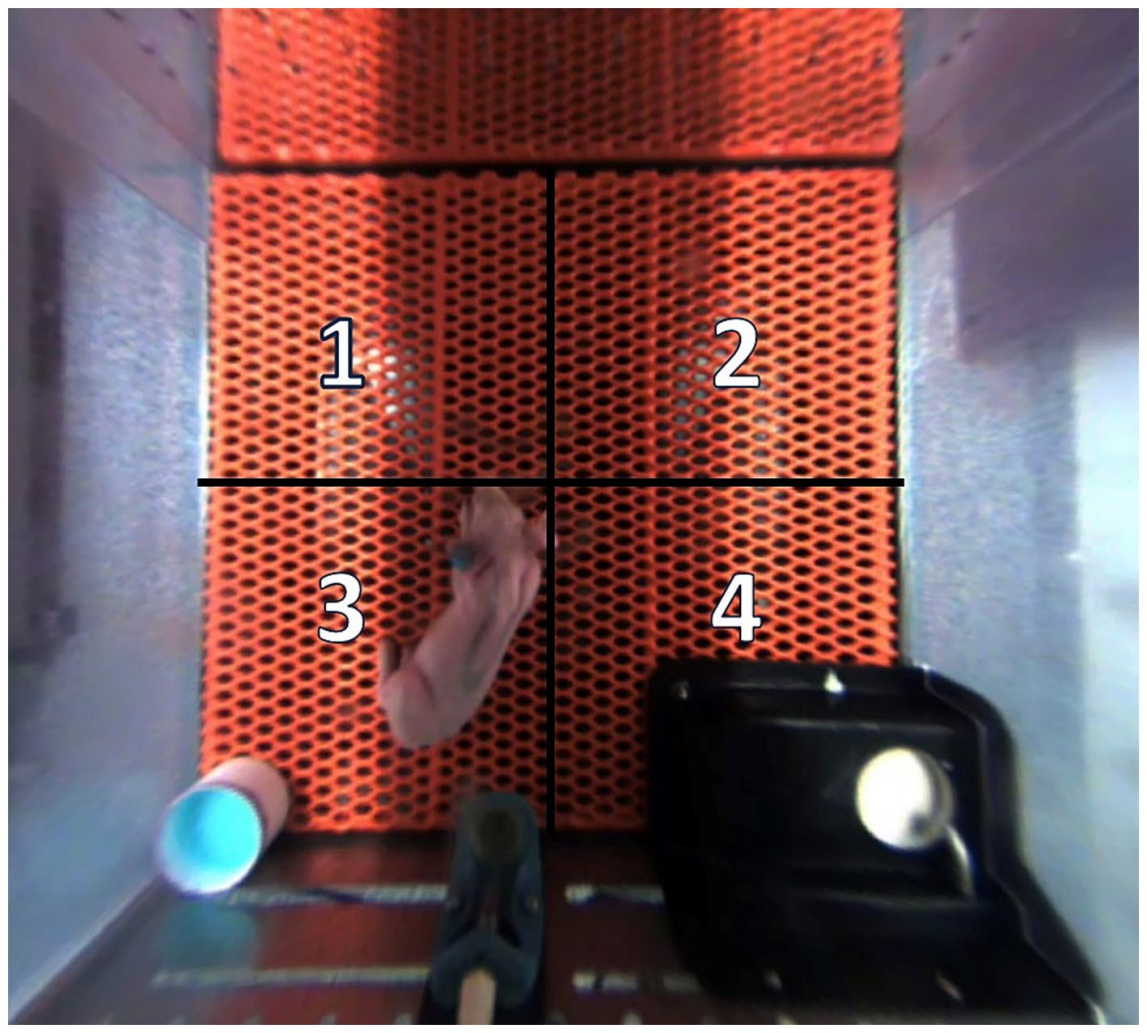
Quadrant location and orientation within each home-cage. Quadrants 1 and 2 allowed adjacent pigs to socialize through a clear, perforated, acrylic divider. Quadrant 3 housed an electrolyte bowl for the first 5 d of the study, and this is the same quadrant where pigs typically defecate and urinate. Quadrant 4 housed the feeding bowl. Pigs were artificially reared from postnatal day (PND) 2–28, dosed with dextran sodium sulfate (DSS) on PND 14–18, and euthanized on PND 27 or 28 to permit sample collection.

After image processing was complete (i.e., coordinates were generated), the data were analyzed through a series of custom scripts that consolidated coordinate data into a condensed format. A quadrant-based division of the home-cage camera view was then applied to segregate 4 areas of approximately equal area. Subsequently, each pig’s individual (x, y) coordinate information was associated with a specific home-cage quadrant (Figure 1), thereby providing an understanding of the pig’s location and time spent within each quadrant in the home-cage context throughout the study.

For this study, continuous video recording was analyzed with a frame rate of 20 frames per second. Every 100th frame was selected, extrapolated, and assumed to cover a 5-s period and this selected frame was subsequently converted to represent the total duration spent within each quadrant. Due to data loss within each cage (i.e., frames where the pig was not detected by the tracking algorithm), positional preference within the home-cage was ultimately expressed on a relative basis, where total minutes in each quadrant was expressed as a proportion of total time (% of total) as analyzed for each pig.

### 2.3 Magnetic resonance imaging

On PND 26 or 27, all pigs underwent neuroimaging procedures at the Beckman Institute Biomedical Center using a Siemens MAGNETOM Prisma 3 T MRI. The neuroimaging protocol included three scans: a magnetization-prepared rapid gradient-echo (MPRAGE) sequence to assess brain macrostructure; diffusion tensor imaging (DTI) to assess brain microstructure; and a multicomponent-driven equilibrium single pulse observation of T_1_ and T_2_ (mcDESPOT) sequence to understand myelin-associated water fraction (MWF). Prior to imaging acquisition, pigs were sedated via an intramuscular injection of a telazol:ketamine:xylazine cocktail [50.0 mg tiletamine plus 50.0 mg of zolazepam reconstituted with 2.50 mL ketamine (100 g/L) and 2.50 mL xylazine (100 g/L); Fort Dodge Animal Health, Overland Park, KS] at 0.03 mL/kg of body weight. Once immobilized, each pig was fitted with earplugs, eyes were secured closed with lightweight surgical tape, and pigs were placed in a supine position with their head fitted into a 15-channel knee coil (1 Tx/15 Rx knee coil for Prisma from QED). A mask was fitted onto the pig’s snout to administer inhalation of isoflurane at 2 and 98% oxygen for anesthesia maintenance throughout the duration of the scan. Percent isoflurane, heart rate and partial pressure of oxygen (PO_2_) were monitored and recorded utilizing two infrared sensor pulse oximeters (LifeWindow LW9x, Boynton Beach, FL and MEDRAD Veris 8600, Indianola, PA) clipped onto the pig’s tail and/or hind hoof. Total scan time amounted to approximately 1.25 h per pig.

#### 2.3.1 Structural MRI acquisition and analysis

MPRAGE sequences were obtained from the tip of the snout to the cervical/thoracic spinal cord junction. Sagittal-oriented data was captured with a 173 mm × 173 mm × 153.6 mm field of view (FOV) and a 288 × 288 × 256 matrix. The following specific parameters were utilized: repetition time (TR) = 2,060 ms, echo time (TE) = 2.05 ms, inversion time (TI) = 1,060 ms, and flip angle (α) = 9° providing a final voxel volume of 0.6 × 0.6 × 0.6 mm^3^. Image processing followed previously described methods (Golden et al., 2023).

##### 2.3.1.1 Voxel-based morphometry pre-processing

Utilizing MPRAGE-acquired images, voxel-based morphometry assessment was conducted using SPM12 (Statistical Parametric Mapping; Institute of Neurology, University College London, London, UK) in MATLAB version R2022a (Mathworks Inc.) to obtain gray and white matter concentrations. During segmentation procedures, as described in Golden et al. (2023), “Native + Dartel Imported” was selected for the Native Tissue option for both gray and white matter tissues to create rigidly aligned tissue class images for each subject. Using the Diffeomorphic Anatomical Registration through Exponential Lie Algebra (DARTEL) toolbox, nonlinear deformations were estimated for the produced images using the “Run Dartel (create Template)” module with default options that resulted in a final template of averaged Dartel registered data. Next, Jacobian-scaled warped tissue class images were created for each subject using the “Normalize to MNI Space” module with pig-specific parameters. The “Dartel Template” parameter was left blank to prevent the incorporation of human-specific MNI space. A voxel size of 0.7 mm^3^, bounding box of −30.1 to 30.1, −35 to 44.8, −28 to 31.5, and 4 mm full-width half maximum (FWHM) were specified.

#### 2.3.2 Diffusion tensor imaging acquisition and analysis

To estimate white matter integrity and microstructure, a diffusion-weighted echo planar imaging (DWI-EPI) sequence was utilized (Feinberg et al., 2010; Moeller et al., 2010; Xu et al., 2013). Images were acquired in transverse orientation with a 160 mm × 160 mm × 80 mm FOV and 100 × 100 × 50 matrix size. The following parameters were utilized: TR/TE = 5,100 ms/70.00 ms, α = 90°, GRAPPA accelerated by a factor of 2 in the phase encode direction, multiband factor of 1, and 3 diffusion weightings at 0, 1,000 and 2,000 s/mm^2^ across 30 directions.

The preprocessing of DTI data involved several steps. Raw DWI images were first denoised using the Marchenko-Pastur Principal Component Analysis (MPPCA) method (Manjón et al., 2013; Neto Henriques, 2018; Veraart et al., 2016a; Veraart et al., 2016b) within the DIPY toolbox (Garyfallidis et al., 2014). Following denoising, between-volume motion correction was performed using an affine registration method (Jenkinson and Smith, 2001), also implemented through DIPY. This step corrects for subject movement between different DWI volumes, subsequently reducing motion artifacts. Additionally, manual whole brain tissue segmentation was performed by trained technicians for the delineation of brain structures.

Diffusion tensors were reconstructed using the diffusion model (Basser et al., 1994; Pajevic and Pierpaoli, 1999) as provided by DIPY. This reconstruction produces maps of fractional anisotropy (FA), mean diffusivity (MD), axial diffusivity (AD), and radial diffusivity (RD), which were used to assess microstructural properties of white matter. Affine registration was conducted using ANTs (Avants et al., 2014). Each subject’s FA map was registered to their corresponding MPRAGE image, allowing regions of interest (ROI) defined in MPRAGE space to be warped to diffusion space. This registration facilitated the extraction of mean FA, MD, RD, and AD values from the ROIs for subsequent statistical analysis.

#### 2.3.3 Myelin water fraction acquisition and analysis

An established form of myelin water imaging, called multicomponent-driven equilibrium single pulse observation of T_1_ and T_2_ (mcDESPOT) (Deoni et al., 2008; Deoni, 2011; Deoni and Kolind, 2015) was conducted for myelin content estimation in young pigs. Utilizing multiple sets of spoiled gradient-recalled echo (SPGR) and T_1_/T_2_-weighted balanced steady-state free precession (SSFP) acquired at varying flip angles (α) and with a constant TR, estimates were derived for various parameters. These parameters included T_1_, T_2_, water residence times, and water volume fractions for the extracellular and intracellular pools of water that comprise the lipid bilayers of myelin.

Sagittal-oriented images were acquired with a 160 mm × 160 mm × 124.8 mm FOV and 128 × 128 × 96 matrix. SSFP data were acquired with two phase-cycling increments of 0° and 180° utilizing the following parameters: TR/TE = 5.3 ms/2.7 ms, α = (11, 15, 19, 23, 27, 35, 50, and 70)°, and bandwidth = 350 Hz/Px, providing a final voxel volume of 1.3 × 1.3 × 1.3 mm^3^. On the other hand, SPGR data were acquired with the following parameters: TR/TE = 5.6 ms/2.7 ms, α = (3, 4, 5, 6, 7, 9, 13, and 18)°, and bandwidth = 350 Hz/Px, providing a final voxel volume of 1.3 × 1.3 × 1.3 mm^3^. To correct for transmit (B_1_) magnetic field inhomogeneities, an additional two high resolution T_1_-weighted inversion recovery (IR)-SPGR sequences were acquired with inversion times of 450 and 750 ms. IR-SPGR parameters were as follows: TR/TE = 5.6 ms/ 2.7 ms, α = 5°, and bandwidth = 350 Hz/Px, providing a final voxel volume of 1.7 × 1.7 × 2.6 mm^3^. Image processing followed previously described methods for pigs at 4-weeks of age (Golden et al., 2023).

### 2.4 Statistical analysis

All outcomes were analyzed by a one-way analysis of variance (ANOVA) using the MIXED procedure in SAS (RRID: SCR_008567; version 9.3; SAS Inst. Inc., Cary, NC, United States). For tracking data, analysis was performed across treatments for each quadrant within each defined study period. The main effect of treatment was assessed individually within each brain region of interest. Cohort was included as a random effect to control for variance between replicates of pigs. Outliers were determined and removed when studentized residuals exceeded ±3 . The level of significance was set to *p <* 0.05.

#### 2.4.1 Voxel-based morphometry statistical analysis

To compare modulated gray and white matter concentrations across the three treatment groups, the statistical non-parametric methods (SnPM) toolbox was utilized.^1^ Data were subjected to a one-way ANOVA specifying an absolute threshold of 0.2 and ANCOVA global normalization. Using the Dartel-generated final template as an inclusive mask, an uncorrected *p* < 0.01 and a threshold of 20 edge-connected voxels were applied.

## 3 Results

### 3.1 Home-cage tracking

Results for home-cage tracking are displayed in Table 1. There were no differences in time spent within quadrants prior to DSS administration or when analyzed during the overall study period. However, after initiating the DSS dosing period, pigs in the CON treatment spent more (*p* < 0.05) time within quadrant 4 than TBCD+DSS pigs, with the DSS treatment being intermediate.

**Table 1:** Effects of orally supplemented TBCD and DSS-induced colitis on relative time spent in each home-cage quadrant^1^.

Outcome per study period	Treatment			Pooled SEM	*p*-value
	Control	DSS	TBCD + DSS		
Pigs, *n*	7	7	6	–	–
**Before DSS (PND 3–13)**					
Quadrant 1	40.62	45.78	27.64	7.269	0.19
Quadrant 2	48.45	41.46	58.76	7.496	0.26
Quadrant 3	3.18	3.17	4.54	1.120	0.58
Quadrant 4	7.74	9.59	9.06	2.003	0.78
**After DSS (PND 14–27/28)**					
Quadrant 1	25.75	23.56	22.85	8.036	0.96
Quadrant 2	35.23	44.02	56.81	7.426	0.11
Quadrant 3	12.89	10.68	6.83	2.812	0.28
Quadrant 4	26.13^a^	21.73^ab^	13.50^b^	3.590	0.046
**Overall (PND 3–27/28)**					
Quadrant 1	32.19	33.67	24.32	6.457	0.52
Quadrant 2	41.42	42.40	57.23	6.162	0.12
Quadrant 3	8.60	7.39	6.82	2.158	0.82
Quadrant 4	17.79	16.54	11.62	2.333	0.14

1 Pigs were artificially reared from postnatal d 2–28, dosed with DSS on postnatal d 14–18, and euthanized on postnatal d 27 or 28 to permit sample collection.

^ab^Means lacking a common superscript letter within a row differ (*p* < 0.05).

DSS, dextran sodium sulfate; PND, postnatal day; TBCD, gamma-cyclodextrin-encapsulated tributyrin.

### 3.2 MRI outcomes

A total of 24 pigs successfully underwent MRI procedures. Across MRI outcomes, replication between treatment groups differs due to outlier removal or image processing issues. As such, final sample sizes are listed in each corresponding outcome table and figure.

#### 3.2.1 Volumetric and voxel-based morphometry outcomes

**Figure 2:**
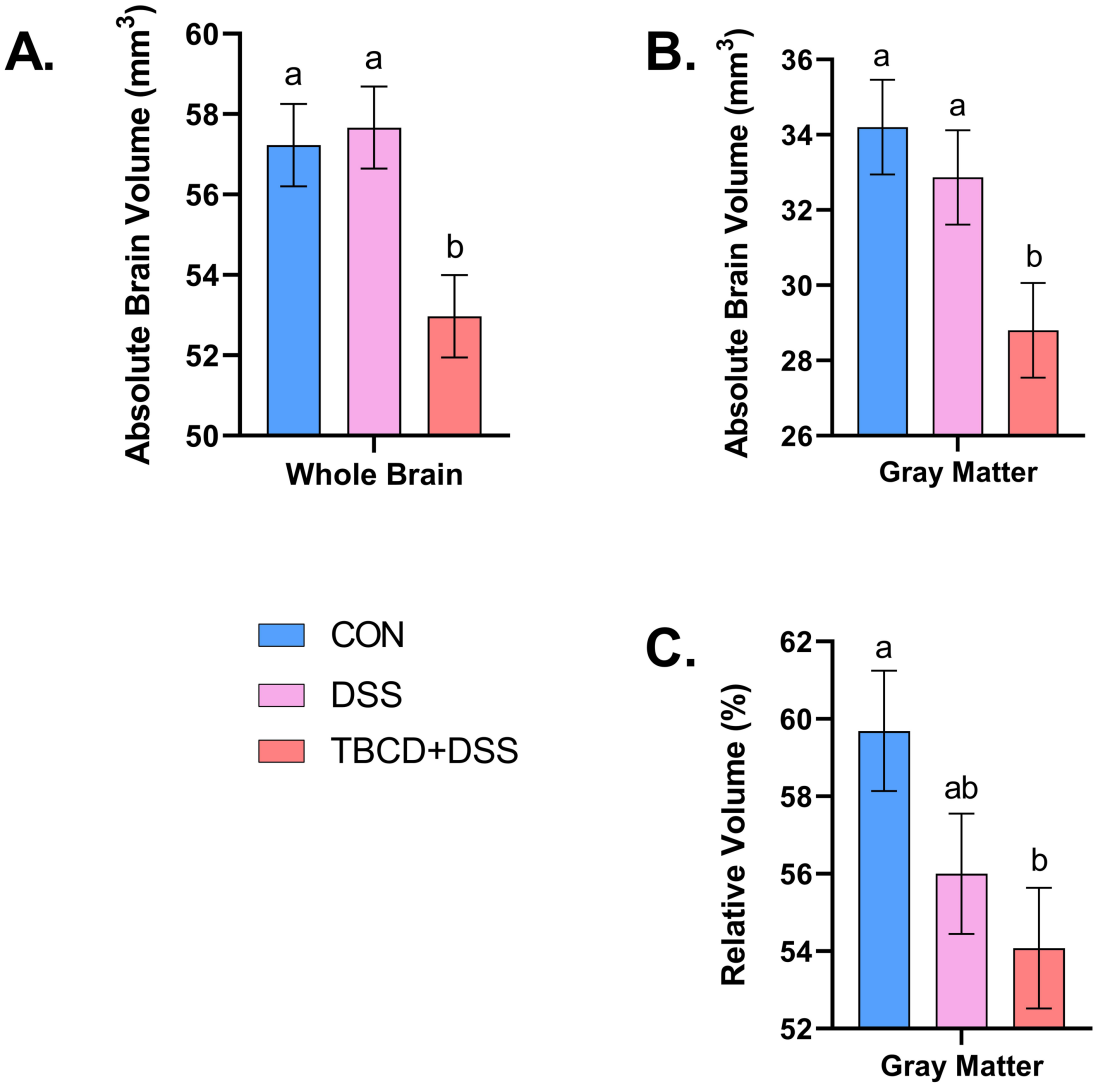
Absolute/relative whole brain and gray matter volumes. **(A)** Absolute volume of the whole brain is displayed for each treatment group (overall treatment effect; *p* = 0.006). The TBCD+DSS group ( $n=8;\mu =52,974\;{\mathrm{mm}}^{3}$ ) was observed to have the smallest absolute volume compared with both the CON ( $n=8;\mu =57,230\;{\mathrm{mm}}^{3}$ ) and DSS ( $n=7;\mu =57,666\;{\mathrm{mm}}^{3}$ ) groups. **(B)** Differences (*p =* 0.013) were observed for absolute gray matter volume. Similarly, the TBCD+DSS group ( $n=8;\mu =28,802\;{\mathrm{mm}}^{3}$ ) was observed to have the smallest absolute volume compared with both the CON ( $n=8;\mu =34,206\;{\mathrm{mm}}^{3}$ ) and DSS ( $n=7;\mu =32,867\;{\mathrm{mm}}^{3}$ ) groups. **(C)** Relative volume of gray matter is displayed for each treatment group (overall treatment effect; *p* = 0.041). The TBCD+DSS group ( n=8;μ=54.08% ) was observed to have a smaller volume compared with the CON group ( n=8;μ=59.69% ), with the DSS group ( n=7;μ=56.88% ) as intermediary. Abbreviations: CON, treatment group given a control diet; DSS, treatment group given the control diet and orally dosed with dextran sodium sulfate; TBCD+DSS, treatment group supplemented with 8.3 g/kg of gamma-cyclodextrin-encapsulated tributyrin and orally dosed with DSS. ^ab^Means lacking a common superscript letter differ (*p* < 0.05).

Differences in absolute volume were observed for the whole brain and gray matter (*p* < 0.05) (Figure 2). Pigs in the TBCD+DSS treatment group had smaller absolute whole brain (*p* = 0.006) and gray matter (*p* = 0.013) volumes compared with both the CON and DSS treatments. Differences in absolute volume were also observed for 28 of the 29 isolated ROI (*p <* 0.05). A similar effect was observed where pigs in the TBCD+DSS group had lower (*p <* 0.05) absolute ROI volumes compared with both other treatments. Absolute brain volumes are displayed in Supplementary Table S1.

A difference in relative volume was only observed for gray matter (*p* = 0.041) (Figure 2). Pigs in the TBCD+DSS group were observed to have a lower (*p <* 0.05) relative volume of gray matter compared with the CON group. No other relative volume differences were observed. All relative volume results are displayed in Table 2. Moreover, voxel-based morphometry analyses revealed no significant cluster differences across treatment groups for either gray or white matter.

**Table 2:** Effects of orally supplemented TBCD and DSS-induced colitis on relative brain volumes (% of total brain volume) of young pigs^1^.

	Treatment			Pooled SEM	*p*-value
ROI	Control	DSS	TBCD + DSS		
*n*	8	7	8	–	–
Gray matter	59.69^a^	56.88^ab^	54.08^b^	1.555	0.041
White matter	27.79	27.50	30.12	0.807	0.067
Cerebral spinal fluid	13.89	15.80	14.83	1.060	0.209
Cerebellum	11.97	11.88	12.00	0.082	0.533
Cerebral aqueduct	0.041	0.041	0.041	0.001	0.942
Corpus callosum	0.462	0.468	0.461	0.005	0.339
Fourth ventricle	0.073	0.074	0.073	0.001	0.974
Hypothalamus	0.166	0.166	0.165	0.001	0.744
Lateral ventricle	0.578	0.578	0.568	0.007	0.274
Left caudate	0.480	0.481	0.473	0.005	0.356
Left cortex	33.40	33.11	33.24	0.207	0.561
Left hippocampus	0.635	0.627	0.631	0.004	0.309
Left inferior colliculus	0.152	0.151	0.153	0.001	0.465
Left internal capsule	0.816	0.813	0.813	0.005	0.757
Left olfactory bulb	2.007	2.009	2.011	0.016	0.983
Left putamen-globus pallidus	0.230	0.227	0.227	0.002	0.225
Left superior colliculus	0.288	0.286	0.288	0.002	0.545
Medulla	3.129	3.102	3.131	0.023	0.591
Midbrain	3.823	3.802	3.832	0.024	0.637
Nucleus accumbens	0.045	0.045	0.045	0.001	0.953
Pons	2.410	2.401	2.404	0.015	0.865
Putamen	0.475	0.473	0.471	0.003	0.303
Right caudate	0.461	0.458	0.456	0.004	0.258
Right cortex	32.04	31.84	31.90	0.195	0.649
Right hippocampus	0.656	0.649	0.654	0.004	0.414
Right inferior colliculus	0.156	0.157	0.158	0.001	0.799
Right internal capsule	0.909	0.909	0.909	0.006	0.990
Right olfactory bulb	2.033	2.026	2.029	0.014	0.883
Right putamen-globus pallidus	0.246	0.246	0.245	0.002	0.517
Right superior colliculus	0.292	0.291	0.290	0.002	0.835
Substantia nigra	0.032	0.032	0.032	0.001	0.676
Thalamus	2.334	2.325	2.330	0.020	0.933

1 Data presented are least squares means and *p*-values from mixed model 1-way ANOVA.

^ab^Means lacking a common superscript letter within a row differ (*p* < 0.05).

DSS, dextrin sodium sulfate; TBCD, cyclodextrin-encapsulated tributyrin; ROI, region of interest; SEM, standard error of the mean.

#### 3.2.2 Diffusion tensor imaging outcomes

**Figure 3:**
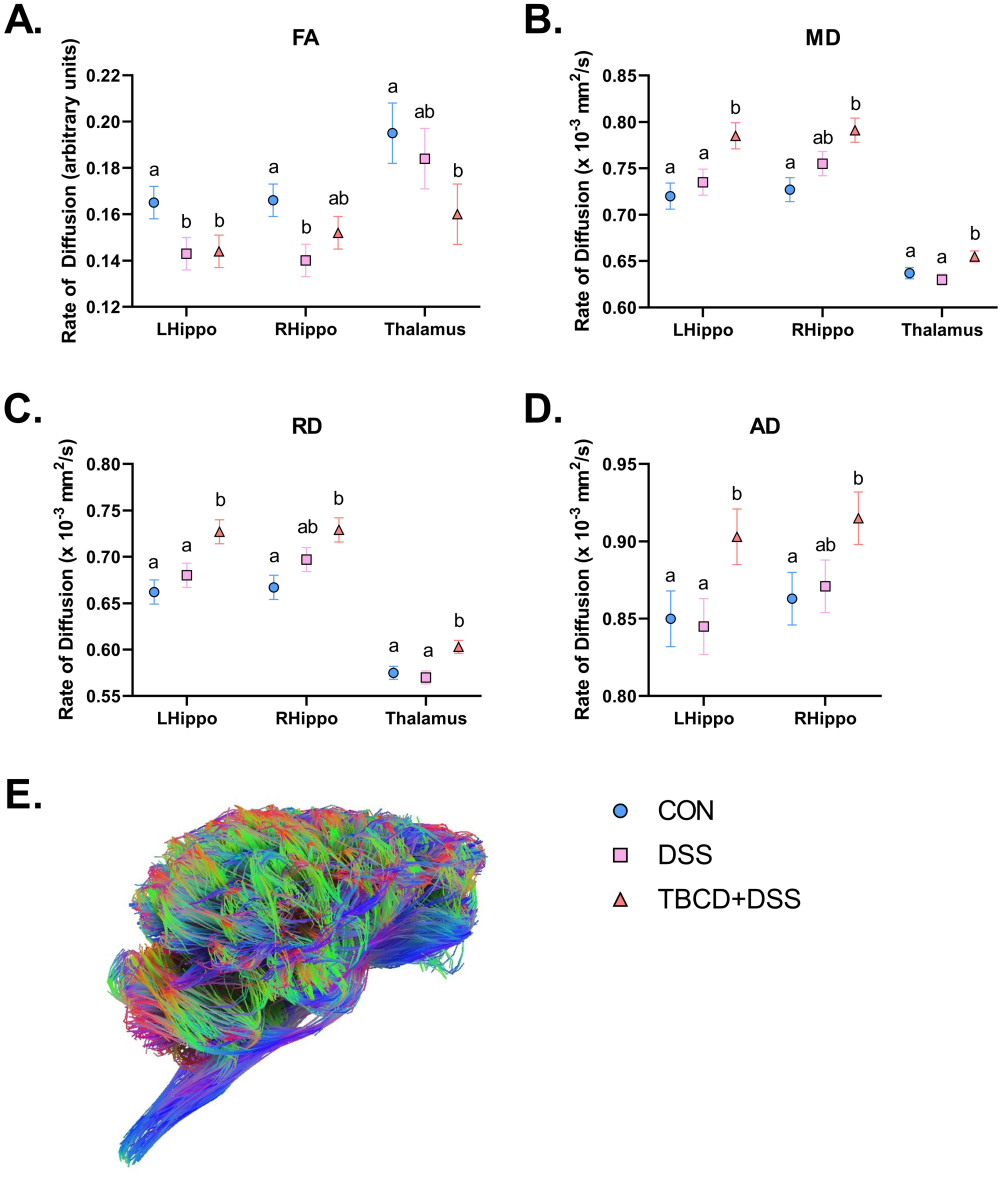
Diffusion tensor imaging results. Refer to Table 3 for specific values. **(A)** Fractional anisotropy (FA) results displayed for the left/right hippocampi and thalamus. For the left hippocampus, both the DSS group and TBCD+DSS groups were observed to have lower values compared with CON. For the right hippocampus, the DSS group was observed to have lower values compared with CON, with TBCD+DSS as intermediary. For the thalamus, the TBCD+DSS group was observed to have lower values compared with CON, with DSS as intermediary. **(B)** Mean diffusivity (MD) results displayed for the left/right hippocampi and thalamus. For both the left hippocampus and thalamus, the TBCD+DSS group was observed to have higher values compared with both DSS and CON groups. For the right hippocampus, the TBCD+DSS group had higher values compared with CON, and DSS as intermediary. **(C)** Radial diffusivity (RD) results are displayed for the left/right hippocampi and thalamus. Similar to MD for the left hippocampus and thalamus, the TBCD+DSS group was observed to have the highest values compared with both other treatment groups. For the right hippocampus, RD was higher for the TBCD+DSS group compared with the CON, with DSS as intermediary. **(D)** Axial diffusivity (AD) results are displayed for the left/right hippocampi. For the left hippocampus, AD values were highest in the TBCD+DSS group compared with both other treatment groups. In the right hippocampus, the TBCD+DSS group had higher AD compared with CON, and DSS as intermediary. **(E)** A 3D reconstructed fiber tractography image is displayed for a representative TBCD+DSS pig. The various colors correspond to the predominant direction of fibers across the brain. Red fibers correspond to transverse fibers, green corresponds to anterior–posterior fibers, and blue fibers correspond to superior–inferior fibers. Fibers that have oblique orientation are represented with the combination of these colors (red + blue = magenta; green + red = yellow; green + blue = cyan). Abbreviations: CON, treatment group given a control diet; DSS, treatment group given the control diet and orally dosed with dextran sodium sulfate; TBCD+DSS, treatment group supplemented with 8.3 g/kg of gamma-cyclodextrin-encapsulated tributyrin and orally dosed with DSS. ^ab^Means lacking a common superscript letter differ (*p* < 0.05).

Across the four diffusion measures, several regional differences were observed between treatment groups (Table 3; Figure 3). For fractional anisotropy, these regions included: hypothalamus (*p* = 0.025), left and right hippocampus (*p* = 0.034 and 0.008, respectively), right internal capsule (*p* = 0.029), and thalamus (*p* = 0.040). For the hypothalamus and thalamus, the CON group had higher (*p <* 0.05) FA values compared with the TBCD+DSS group, with the DSS group as intermediary. For the left hippocampus, the CON group had higher (*p <* 0.05) FA compared with both DSS and TBCD+DSS treatment groups. In the right hippocampus, the same effect was observed although DSS was intermediary. In the right internal capsule, the CON and DSS groups had larger (*p <* 0.05) FA values compared with the TBCD+DSS group.

**Table 3:** Effects of orally supplemented TBCD and DSS-induced colitis on DTI measurements in the brain of young pigs^1^.

	Treatment			Pooled SEM	*p*-value
ROI	Control	DSS	TBCD + DSS		
*n*	9	6	8	–	–
**Fractional anisotropy**					
Left hippocampus	0.165^a^	0.143^b^	0.144^b^	0.007	0.034
Right hippocampus	0.166^a^	0.140^b^	0.152^ab^	0.007	0.008
Thalamus	0.195^a^	0.184^ab^	0.160^b^	0.013	0.040
Right internal capsule	0.401^a^	0.397^a^	0.316^b^	0.028	0.029
Hypothalamus	0.177^a^	0.165^ab^	0.148^b^	0.009	0.025
**Mean diffusivity**					
Left hippocampus	0.720^a^	0.735^a^	0.785^b^	0.014	0.003
Right hippocampus	0.727^a^	0.755^ab^	0.791^b^	0.013	0.005
Thalamus	0.637^a^	0.630^a^	0.655^b^	0.006	0.016
**Radial diffusivity**					
Left hippocampus	0.662^a^	0.680^a^	0.727^b^	0.013	0.002
Right hippocampus	0.667^a^	0.697^ab^	0.729^b^	0.013	0.003
Thalamus	0.575^a^	0.570^a^	0.603^b^	0.007	0.005
Medulla	0.364^a^	0.396^ab^	0.447^b^	0.020	0.014
Left superior colliculus	0.547^a^	0.580^ab^	0.637^b^	0.022	0.015
Right cortex	0.630^a^	0.664^ab^	0.668^b^	0.016	0.050
Right internal capsule	0.430^a^	0.447^ab^	0.498^b^	0.022	0.047
Pons	0.439^a^	0.419^a^	0.496^b^	0.022	0.039
**Axial diffusivity**					
Left hippocampus	0.850^a^	0.845^a^	0.903^b^	0.018	0.035
Right hippocampus	0.863^a^	0.871^ab^	0.915^b^	0.017	0.049
Medulla	0.534^a^	0.596^ab^	0.648^b^	0.023	0.004
Corpus callosum	0.971^ab^	1.020^a^	0.900^b^	0.032	0.030

1 Data presented are least squares means and *p*-values from mixed model 1-way ANOVA.

^ab^Means lacking a common superscript letter within a row differ (*p* < 0.05).

DTI; diffusion tensor imaging; DSS, dextrin sodium sulfate; TBCD, cyclodextrin-encapsulated tributyrin; ROI, region of interest; SEM, standard error of the mean.

Conversely, in some regions where FA was observed to be higher for a treatment, across the other diffusion metrics (MD, RD, and AD) values were lower for that same treatment (displayed in Table 3; Figure 3). For example, in the left hippocampus, across all three DTI measures, the CON and DSS groups had lower (*p* < 0.05) values compared with the TBCD+DSS group (*p* < 0.05). Similarly, the same effect was observed in the thalamus for MD (*p* = 0.016) and RD (*p* = 0.005). In the right hippocampus, the CON group had lower values compared with the TBCD+DSS group, with the DSS group as intermediary for MD (*p* = 0.005), RD (*p* = 0.003), and AD (*p* = 0.049). Several additional regions had a treatment effect for RD. In the medulla, left superior colliculus, right cortex, and right internal capsule, the CON group was observed to have lower (*p* < 0.05) RD compared with the TBCD+DSS group, with DSS as intermediary. In the pons, both CON and DSS groups had lower (*p* < 0.05) RD compared with the TBCD+DSS group. Contrary to other measures, AD in the corpus callosum had a different trend, where the DSS group had higher (*p* = 0.03) AD compared with the TBCD+DSS group.

#### 3.2.3 Myelin water fraction imaging outcomes

**Figure 4:**
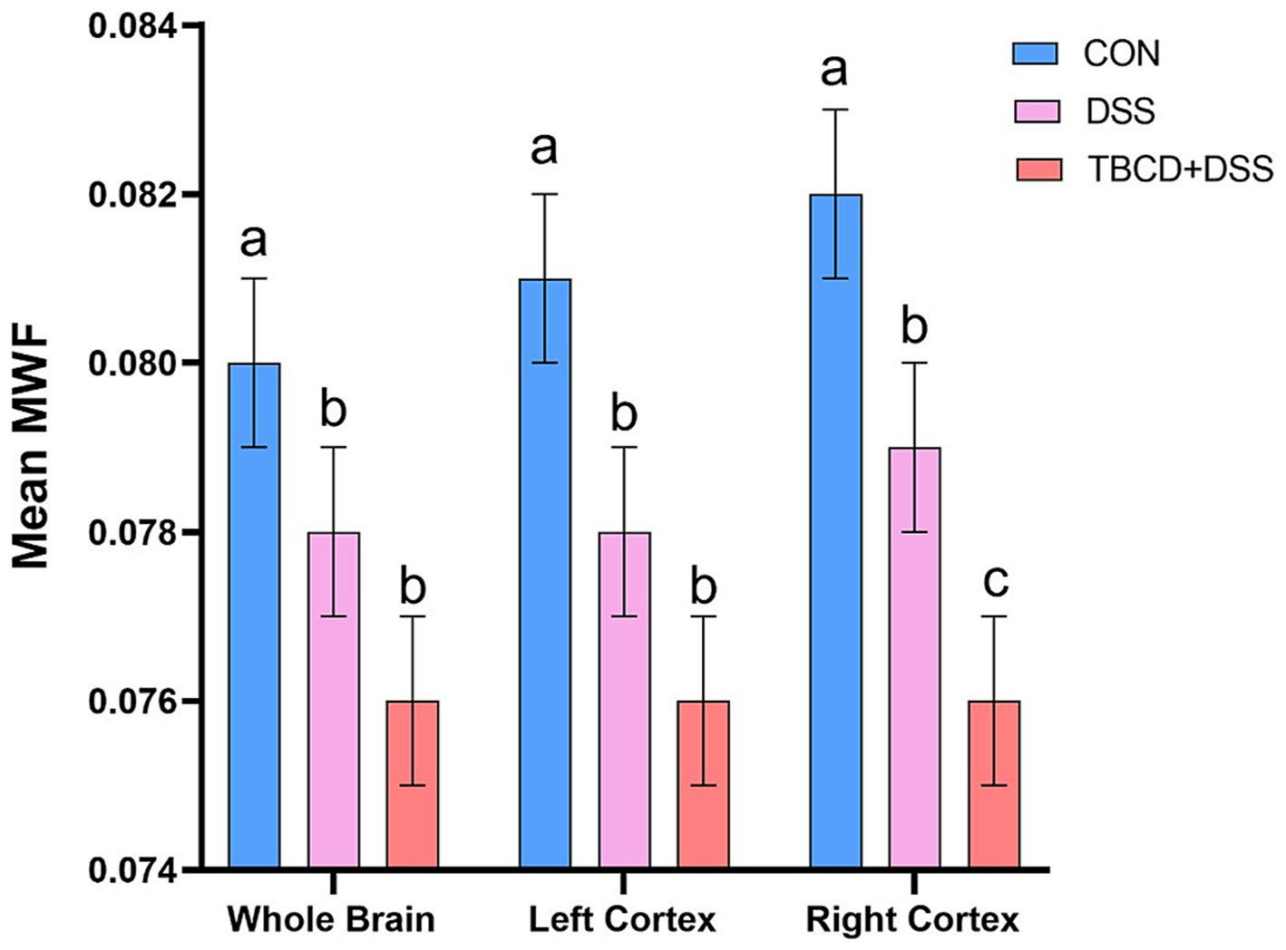
Mean myelin water fraction (MWF) results*. For the whole brain and left cortex, mean MWF values were lower in the DSS $(n=7;\mu_{WB}=0.078;\mu_{LC}=0.078$ ) and TBCD+DSS $(n=7;\mu_{WB}=0.076;\mu_{LC}=0.076$ ) groups compared with the CON group $(n=7;\mu_{WB}=0.080;\mu_{LC}=0.081$ ). For the right cortex, all treatment groups differed from each other. Mean MWF was lowest in the TBCD+DSS group (n=7;μ=0.076 ), followed by the DSS group $\left(n=7;\mu =0.079\right)$ , with the highest mean in the CON group (n=7;μ=0.082 ). Abbreviations: CON, treatment group given a control diet; DSS, treatment group given the control diet and orally dosed with dextran sodium sulfate; TBCD+DSS, treatment group supplemented with 8.3 g/kg of gamma-cyclodextrin-encapsulated tributyrin and orally dosed with DSS; $\mu_{LC}$ , left cortex mean MWF; $\mu_{WB}$ , whole brain mean MWF. *Mean MWF is displayed in arbitrary units. ^ab^Means lacking a common superscript letter differ (*p* < 0.05).

Differences in mean myelin water fraction were observed for the whole brain and both left and right cortices (Figure 4). For overall mean myelin water fraction in the whole brain (*p =* 0.003) and left cortex (*p =* 0.001), pigs supplemented with DSS (DSS and TBCD+DSS treatments) had less myelin compared with the CON pigs. For the right cortex, all three treatments differed (*p =* 0.001) from each other. The TBCD+DSS had the lowest (*p* < 0.05) mean MWF, whereas CON pigs had the highest (*p* < 0.05) value overall. MWF values for all other regions are listed in Supplementary Table S2.

## 4 Discussion

Colitis is a chronic disease with unclear etiology and no established cure. Additionally, with the wide range of extraintestinal manifestations associated with colitis and potential negative effects on brain functional connectivity (Kornelsen et al., 2021; Scheid and Teich, 2007; Wang et al., 2022), investigating modes of action and potential ameliorating mechanisms is of utmost importance. In the present study, the effects of experimentally induced colitis and TBCD supplementation on home-cage behavior and brain structure of young pigs were investigated. Representative neuroimaging was conducted to investigate influences on brain macrostructure, microstructure, and myelin quantification. To our knowledge, this is the first study to investigate how brain development may be altered in a colitis model using domestic pigs. This study is in conjunction with Sommer et al. (2022), which previously reported growth performance, intestinal outcomes, and serum cytokine concentrations (IL-1β, IL-6, and TNFα) associated with the DSS-induced colitis model in pigs.

### 4.1 Home-cage tracking

Pigs underwent video tracking analysis to discern how experimentally induced colitis and TBCD supplementation altered innate behaviors within the pig’s home-cage. Prior to DSS administration and during the overall study, pigs within all treatments spent a similar amount of time within each quadrant located near the clear, perforated divider between adjacent pigs on a given level (i.e., quadrants 1 and 2). Furthermore, pigs spent the least amount of time (3–8%) within the quadrant where defecation and urination typically occur (quadrant 3), while they spent the rest of their time within the quadrant that houses the milk bowl.

Following DSS administration, pigs in the TBCD+DSS treatment spent less time within the quadrant that houses the milk bowl compared with CON pigs, thereby indicating that TBCD supplementation altered the spatial preference of pigs undergoing an experimentally induced colitis challenge. This decrease in time spent within the quadrant housing the milk bowl further supports findings reported by Sommer et al. (2022), which indicated that pigs receiving supplemental TBCD consumed less milk replacer after DSS dosing was initiated. This is contrary to previous work completed in mice and pigs, which indicated that DSS administration decreased milk intake (Bamba et al., 2012; Nielsen et al., 2020). In the present study, TBCD+DSS pigs consumed less milk replacer, though that was not the case for DSS pigs (Sommer et al., 2022), thereby suggesting potential interactive effects of TBCD supplementation during DSS-induced colonic inflammation. Similar intake results were reported by Weber and Kerr (2008), where linear increases in sodium butyrate supplementation in weaned pigs resulted in decreased feed intake. This decrease in milk intake and time spent within the quadrant housing the milk bowl may indicate that spatial preference correlates to specific activities within the home-cage, such as food aversion, sleeping, or urination, though further analysis of this phenomenon is needed.

### 4.2 Magnetic resonance imaging

#### 4.2.1 Macrostructure assessment

For general assessment of region-specific volumes, anatomical images were acquired to calculate absolute and relative volume of whole brain, white matter, gray matter, cerebral spinal fluid and 29 regions of interest. Across multiple brain regions, lower absolute volumes were observed for the TBCD+DSS group. This finding can be explained by growth performance results reported by Sommer et al. (2022), where it was observed that TBCD+DSS pigs had overall decreased body weight from PND 15 to study conclusion compared with both other treatment groups. With a smaller body size in general, it is expected that absolute volume of the brain and corresponding regions would be smaller.

Once volume was considered relative to whole brain size, most treatment effects dissipated. This finding provides support that allometric brain growth was largely unaltered by treatment group, other than for relative gray matter volume. Contrary to previous work by Kornelsen et al. (2021), we observed lower relative gray matter volume in the TBCD+DSS group and no differences between the CON and DSS groups. Kornelsen et al. (2021) conducted VBM analyses and identified several gray matter clusters where patients with ulcerative colitis exhibited larger gray matter volumes compared with healthy controls. On the other hand, Zikou et al. (2014) observed decreased gray matter clusters across several brain regions in patients diagnosed with CD or UC compared with controls. These contradicting results differ from the present study where no significant VBM cluster differences were identified across treatment groups. In general, given the conflicting evidence, it is unclear what to expect with brain volume in a colitis condition, hence bolstering our justification for use of multiple neuroimaging modalities to improve interpretation of results. Therefore, investigating microstructure in conjunction with myelination specific measures was advantageous.

#### 4.2.2 Microstructure assessment

To investigate microstructural organization of the brain, DTI imaging was conducted. This imaging technique captures the diffusion of water molecules across imaging voxels and is used to construct four maps (FA, MD, RD, and AD) that correspond to diffusivity across fibers (Alexander et al., 2007; Chang et al., 2017). Using RD and AD maps, respective parallel and perpendicular water movement across the long axis of fibers can be captured, whereas overall average diffusivity is reflected by MD. During brain development, the tensor parameter that is of most interest is FA, which captures the orientation-specific diffusivity of water molecules within a tissue (Alexander et al., 2007; Chang et al., 2017). In general, FA can be influenced by a variety of microstructural features such as myelination, axon diameter, and axon packing density (Jones et al., 2013), and FA is repeatable and highly sensitive to neurodevelopmental status. Previously, an inverse correlation was observed for FA and RD regarding myelination (Chang et al., 2017; Scheel et al., 2013). Chang et al. (2017) observed that myelin basic protein (MBP) immunofluorescence was positively correlated with FA and negatively correlated with RD, but this dual relationship was only observed in select regions like the corpus callosum, fimbria, and anterior commissure. In other white matter regions, MBP immunofluorescence was only correlated with FA (Chang et al., 2017), highlighting the possibility of FA as a more myelin-specific measure. Even given this evidence, there is much debate on the true biological relevance of the various diffusion parameters (Jones et al., 2013). Specifically, DTI provides an estimate of fiber orientation rather than a precise measurement and parameter values can be highly influenced by crossing fibers (Jeurissen et al., 2013; Jones et al., 2013).

Several trends were observed across diffusion outcomes in the present study. In regions such as the hippocampus, FA was decreased in pigs administered DSS as compared with control pigs. However, the opposite was observed in the other diffusion outcomes, where MD, RD, and AD were increased in both DSS groups compared with control pigs. Similarly, when FA was decreased in the thalamus for the TBCD+DSS group compared with the control, the opposite directional trend was observed for MD, RD, and AD, indicating more restrictions to diffusion. These directional changes align with a previous study by Hou et al. (2020) in a group of CD patients. Patients diagnosed with CD had decreased FA paired with increased MD, RD, and AD in a subset of brain regions when comparing to results in healthy controls (Hou et al., 2020). On the other hand, work in an adolescent population with pediatric onset of CD detected higher FA in several regions and lower MD across multiple cortices in the CD group (Filip et al., 2024). However, this study was completed in CD adolescent patients in remission which may have impacted results.

In our pig study, several other regions were identified as having significant differences across the diffusion parameters, but due to inconsistent differences observed across diffusion parameters, these outcomes were more difficult to interpret. Interestingly, in the corpus callosum, a highly myelinated structure, AD was observed to be lower for the TBCD+DSS group compared with the DSS group, which was not consistent across other regions. These findings are similar to those of Zikou et al. (2014) who observed decreased AD in patients with CD and UC, although lower AD was not observed in pigs administered DSS in the current study. Not all DTI trends were consistent with previous work, which may be due to differences in age. In the present study, young pigs were chosen to represent a pediatric population whereas in human studies it is typical to recruit adult patients with chronic CD and UD (Hou et al., 2020; Zikou et al., 2014).

In general, it is possible these results reflect variance in how axons are distributed within a voxel across treatments (i.e., the architectural paradigm), which may affect overall tissue anisotropy (Jones et al., 2013). Additionally, across development, FA has been shown to steadily increase into adolescence (Barnea-Goraly et al., 2005), which follows the trend for myelination to increase throughout childhood (Croteau-Chonka et al., 2016). Still, the interpretation of DTI parameters is highly debatable, as studies have shown contradicting evidence when it comes to mapping longitudinal development (Arshad et al., 2016). Additionally, metrics like FA can be influenced by a multitude of microstructural features outside of myelination (Jones et al., 2013). Therefore, to gain better insight into how myelination is affected, more myelin specific imaging sequences were acquired.

#### 4.2.3 Myelin assessment

Various myelin-specific neuroimaging methods are available to quantify myelin concentration in the brain. A highly utilized method, specifically in the field of neurodevelopment, is through steady-state-based myelin water imaging (Croteau-Chonka et al., 2016; Dean et al., 2014; Deoni and Kolind, 2015). Myelin water fraction (MWF) can be calculated utilizing the distinct signatures emitted by extracellular vs. intracellular water molecules within the myelin sheath of axons, (Dubois et al., 2014). Generally, studies have shown strong correlations between MWF and myelin histology, justifying it as a potential biomarker of myelin (Lee et al., 2021).

In the present study, across whole brain and both left and right cortices, both DSS groups were identified as having lower MWF values compared with the control group. In particular, the TBCD+DSS group was observed to have the lowest values of MWF in the right cortex. This is relatively consistent with the DTI outcomes that reflected both DSS-treated groups having lower FA values in the hippocampus. Specifically, the TBCD+DSS group was consistently observed to have the lowest FA values across multiple regions other than the right hippocampus. Previously, it has been observed that myelination is a particularly vulnerable process to DSS-induced increased intestinal permeability (Boles et al., 2024) This may explain why colitis has been repeatedly associated with demyelinating disorders such as multiple sclerosis (Scheid and Teich, 2007). Usually, demyelinating conditions manifest after long-term chronic colitis exposure, which has been commonly studied (Scheid and Teich, 2007). However, even acute exposure to DSS has been linked to deficits in recognition memory and anxiety-like behavior in mice several weeks after exposure (Salvo et al., 2020), likely indicating that early-life inflammatory bowel conditions can detrimentally impact brain development. There is limited work on how early-life colitis can impact myelin development, with most early-life colitis research completed in rodents (Boles et al., 2024; Han et al., 2018; Salvo et al., 2020) whereas others indicating no differences in myelination as assessed through neuroimaging in adolescent patients with CD (Filip et al., 2024). Therefore, the findings of this study add to the literature and support the hypothesis that early-life exposure to colitis influences myelination patterns within the developing brain.

### 4.3 Potential mechanisms of action

Previous studies have investigated mechanisms by which DSS-induced colitis impacts the brain. Han et al. (2018) observed increased levels of IL-6 and TNF-ɑ mRNA expression in the brain, suggesting cortical inflammation. Furthermore, they observed downregulated blood–brain-barrier (BBB) tight intracellular junction protein expression and up-regulated cleaved caspase3 expression in the cortex, thereby suggesting changes in BBB permeability and possible apoptosis within the brain of DSS-treated mice (Han et al., 2018). Additionally, colitis has been shown to induce immune activation within the brain (Boles et al., 2024). In a mouse model of DSS colitis, circulating proinflammatory cytokines, such as TNF, IL-10, and LBP, were elevated, which are also elevated in various neuropsychological conditions (Boles et al., 2024). Within the brain, increased expression of lipid oxidation and oxidative stress associated genes (*Alox5*, *Len2*, *Mmp8*, *Nfe2l2*, etc.) were observed, which may be indicative of oxygenation disruption in the brain (Boles et al., 2024). Han et al. (2018) and Boles et al. (2024), both reported elevated circulating cytokine levels, however, DSS-treated piglets in this study did not have increased serum cytokine concentrations at study conclusion (Sommer et al., 2022). These dissimilar findings may be due to species differences or timing of sample collection. Whereas MRI and blood collections occurred a week after the last administration of DSS in pigs, serum and brain samples in mice were collected shortly following the last dosing of DSS (Han et al., 2018). Supporting evidence for the importance of timing was shown by Boles et al. (2024) who identified that severity and symptoms peak immediately after DSS withdrawal. In their mouse colitis model, DSS treatment elevated circulating cytokines 2-days after treatment, which returned to baseline levels within 3 days after the last administration of DSS (Boles et al., 2024).

Additionally, across four different rodent colitis models, Sans et al. (2001) reported upregulation of vascular cell adhesion molecule-1 (VCAM-1) in the brain that was positively correlated with VCAM-1 expression in the colon. VCAM-1 is a cell adhesion molecule that plays a critical role in inflammatory responses, specifically involved in leukocyte emigration to infection sites (Pepinsky et al., 1992). In contrast, Sans et al. (2001) did not observe any leukocyte infiltration into the brain of the rodents with colitis, even with upregulation of VCAM-1, suggesting another mechanism for colitis interaction with the brain.

Though not investigated in the current study, another potential explanation for neurological manifestations of colitis is through links with thrombosis and vasculitis (Saleh et al., 2011; Scheid and Teich, 2007). Inflammatory bowel diseases have been linked with a hypercoagulable state stemming from impaired function of platelets, disturbed coagulation, and fibrinolysis (Twig et al., 2005). Therefore, it has been reported that patients with UC and CD have a higher risk in developing vascular thromboembolism, with UC (1.9%), in particular, having a higher relative risk compared with Crohn’s (1.2%) (Saleh et al., 2011). Moreover, Sheffield et al. (1981) reported occurrence of vascular thromboembolism in rhesus monkeys with UC and found an association with various blood clots in extracerebral veins and demyelinating brain lesions within those same animals. Additionally, cases of cerebral thromboembolism have been reported in pediatric inflammatory bowel disease patients, suggesting that this phenomenon may also occur early in life (Barclay et al., 2010; De Laffolie et al., 2022). Hence, through affecting vasculature, inflammatory bowel conditions, such as colitis, may impact the brain. Despite this, it is important to note that most of this work has been collected using chronic or severely acute inflammatory bowel models, and it is unclear how vasculature is impacted by colitis during development. Further investigation is needed to determine the exact mechanisms of action.

### 4.4 TBCD supplementation

Butyrate is a short chain fatty acid (SCFA) known for its many anti-inflammatory and immunoregulatory effects (Alpino et al., 2024). Additionally, the salt form of butyric acid, sodium butyrate, has been widely used to improve cognitive deficits associated with brain disorders and injury (Alpino et al., 2024; Lynch et al., 2021). Specifically, in a mouse model of traumatic brain injury, supplementation with sodium butyrate ameliorated neurological deficits (Li et al., 2016). Additionally, treatment with sodium butyrate reduced injury-induced BBB permeability (Li et al., 2016). Furthermore, in a cuprizone-induced demyelination mouse model that supplemented with SCFAs, butyrate was specifically shown to ameliorate demyelination (Chen et al., 2019). Chen et al. (2019) also reported that butyrate enhanced the maturation of oligodendrocytes and thereby enhanced remyelination in culture. Although this previous work highlights the beneficial and ameliorative effects of butyrate supplementation, this was not reflected in our pig study.

Contrary to expectations, across all neuroimaging modalities, the TBCD+DSS group consistently had the lowest values compared with both DSS and CON treatment groups. It is possible that the integrity of the encapsulated tributyrin was affected, given the growth performance results reported by Sommer et al. (2022). Due to the organoleptic properties and high rate of absorption of butyrate, maintaining the proper form of tributyrin is necessary to ensure it reaches the colon (Miyoshi et al., 2011; Thibault et al., 2010). Previous work has shown that butyrate supplementation is well tolerated by pigs, although the method of butyrate administration varied (Bartholome et al., 2004). Whereas TBCD was mixed into milk each morning and delivered via an automatic feeding system (Fil et al., 2021) in the current study, Bartholome et al. (2004) utilized a total parenteral nutrition feeding method to administer SCFAs directly into the bloodstream. Others have supplemented tributyrin directly to pigs (Piva et al., 2002), utilized a casein-coated tributyrin (Dong et al., 2016), or implemented a lipid microencapsulation method (Tugnoli et al., 2020). Direct supplementation and coated tributyrin primarily affected the small intestine, whereas encapsulation allowed effects to reach the hindgut (Tugnoli et al., 2020). Therefore, encapsulation is necessary to reach the site of colitis and it is possible that delivery through the automatic feeding system may have impacted the gamma-cyclodextrin encapsulation method utilized since the TBCD+DSS group was reported to have lower feed intake that began during the DSS dosing period (Sommer et al., 2022). Even so, in the current study, delivery of TBCD was deemed successful, as increased butyrate concentrations were observed in the proximal colon of the TBCD+DSS pigs compared with the other treatment groups (Sommer et al., 2022).

Whereas evidence from our pig study supports that oral TBCD supplementation via inclusion in milk replacer was successful, it is possible that the age of the animal could be a factor as previous research has suggested that butyrate supplementation is more effective in older populations (Matt et al., 2018). Specifically, Matt et al. (2018) observed that aged mice exhibited a more anti-inflammatory profile after butyrate supplementation compared with adult mice. Because our study did not include pigs that were supplemented with TBCD in the absence of DSS-inducted colitis, there remain some unanswered questions. Another limitation is that we did not quantify systemic and localized organ butyrate concentrations to identify if and how butyrate supplementation may have impacted the brain. Therefore, future work is warranted to further investigate various supplementation delivery mechanisms and how TBCD supplementation alone impacts brain development.

## 5 Conclusion

Structural brain development was altered in pigs exposed to DSS-induced colitis, and contrary to our hypothesis, TBCD did not ameliorate these effects. Utilizing various neuroimaging modalities, treatment effects were observed that showcased how early-life exposure to colitis influenced myelination. Additionally, home-cage behavior was altered in pigs experimentally induced with colitis and receiving TBCD supplementation. With the expanding prevalence of inflammatory bowel conditions worldwide, this work provides further justification for the importance of investigating pediatric-onset inflammatory bowel diseases.

## Data Availability

The raw data supporting the conclusions of this article will be made available by the authors, without undue reservation.

## Footnotes

^1^ http://nisox.org/Software/SnPM13/
