# Correction: Experimentally induced colitis impacts myelin development and home-cage behavior in young pigs regardless of supplementation with oral gamma-cyclodextrin-encapsulated tributyrin

**DOI:** 10.3389/fnins.2025.1637628

**Published:** 2025-06-11

**Authors:** Loretta T. Sutkus, Kaitlyn M. Sommer, Zimu Li, Bradley P. Sutton, Sharon M. Donovan, Ryan N. Dilger

**Affiliations:** ^1^Neuroscience Program, University of Illinois, Urbana, IL, United States; ^2^Department of Animal Sciences, Division of Nutritional Sciences, University of Illinois, Urbana, IL, United States; ^3^Department of Bioengineering, University of Illinois, Urbana, IL, United States; ^4^Beckman Institute for Advanced Science and Technology, University of Illinois, Urbana, IL, United States; ^5^Department of Food Science and Human Nutrition, University of Illinois, Urbana, IL, United States; ^6^Division of Nutritional Sciences, University of Illinois, Urbana, IL, United States

**Keywords:** brain development, colitis, dextran sodium sulfate, gamma-cyclodextrin encapsulated tributyrin, magnetic resonance imaging

In the published article, an author name was incorrectly written as Sharon D. Donovan. The correct spelling is Sharon M. Donovan.

The authors apologize for this error and state that this does not change the scientific conclusions of the article in any way. The original article has been updated.

