# Supplementary material for: Experimentally induced colitis impacts myelin development and home-cage behavior in young pigs regardless of supplementation with oral gamma-cyclodextrin-encapsulated tributyrin
Source: Front Neurosci. 2025 Mar 31;19:1484497. doi: 10.3389/fnins.2025.1484497 (PMC11994669; doi:10.3389/fnins.2025.1484497)
Supplement: Supplementary file 1 [file Table_1.docx]

| **Supplemental Table 1.** Effects of orally supplemented TBCD and DSS-induced colitis on absolute brain volumes (mm^3^) of young pigs ^1^ | | | | | |
| --- | --- | --- | --- | --- | --- |
|  | **Treatment** | | | **Pooled SEM** | ***P*-Value** |
| **ROI** | **Control** | **DSS** | **DSS+TBCD** |  |  |
| *n* | 8 | 7 | 8 | - | - |
| Whole brain | 57230^a^ | 57666^a^ | 52974^b^ | 1024.0 | **0.006** |
| Gray matter | 34206^a^ | 32867^a^ | 28802^b^ | 1256.97 | **0.013** |
| White matter | 15893 | 15845 | 16404 | 366.76 | 0.470 |
| Cerebral spinal fluid | 7931.7 | 9067.9 | 7836.4 | 581.07 | 0.093 |
| Cerebellum | 6851.7^a^ | 6846.7^a^ | 6362.9^b^ | 139.61 | **0.027** |
| Cerebral aqueduct | 23.2^a^ | 23.4^a^ | 21.5^b^ | 0.55 | **0.037** |
| Corpus callosum | 263.1^a^ | 269.5^a^ | 243.5^b^ | 5.38 | **0.006** |
| Fourth ventricle | 42.0 | 42.5 | 39.0 | 1.13 | 0.068 |
| Hypothalamus | 94.8^a^ | 96.0^a^ | 87.5^b^ | 1.98 | **0.011** |
| Lateral ventricle | 329.3^a^ | 332.6^a^ | 300.4^b^ | 6.89 | **0.005** |
| Left caudate | 273.5^a^ | 276.9^a^ | 250.3^b^ | 5.63 | **0.005** |
| Left cortex | 19100^a^ | 19076^a^ | 17617^b^ | 370.0 | **0.011** |
| Left hippocampus | 363.1^a^ | 361.2^a^ | 334.4^b^ | 7.04 | **0.012** |
| Left inferior colliculi | 87.2^a^ | 87.2^a^ | 80.9^b^ | 1.798 | **0.027** |
| Left internal capsule | 466.2^a^ | 475.3^a^ | 430.2^b^ | 9.858 | **0.008** |
| Left olfactory bulb | 1146.3^a^ | 1157.7^a^ | 1064.3^b^ | 23.53 | **0.017** |
| Left putamen-globus pallidus | 131.2^a^ | 133.3^a^ | 120.1^b^ | 2.958 | **0.009** |
| Left superior colliculi | 164.7^a^ | 167.5^a^ | 152.4^b^ | 3.70 | **0.018** |
| Medulla | 1790.4^a^ | 1788.3^a^ | 1660.3^b^ | 36.80 | **0.026** |
| Midbrain | 2187.4^a^ | 2191.5^a^ | 2031.5^b^ | 43.82 | **0.022** |
| Nucleus Accumbens | 25.7^a^ | 26.1^a^ | 23.9^b^ | 0.618 | **0.034** |
| Pons | 1376.5^a^ | 1367.5^a^ | 1273.3^b^ | 27.84 | **0.023** |
| Putamen | 271.3^a^ | 275.1^a^ | 249.2^b^ | 5.429 | **0.005** |
| Right caudate | 262.2^a^ | 263.5^a^ | 241.0^b^ | 5.187 | **0.008** |
| Right cortex | 18305^a^ | 18339^a^ | 16896^b^ | 353.72 | **0.010** |
| Right hippocampus | 375.2^a^ | 374.1^a^ | 346.8^b^ | 7.431 | **0.017** |
| Right inferior colliculi | 89.4^a^ | 90.2^a^ | 83.5^b^ | 1.792 | **0.024** |
| Right internal capsule | 519.0^a^ | 523.8^a^ | 481.1^b^ | 10.311 | **0.013** |
| Right olfactory bulb | 1160.9^a^ | 1167.1^a^ | 1074.0^b^ | 22.78 | **0.012** |
| Right putamen-globus pallidus | 140.1^a^ | 141.8^a^ | 129.0^b^ | 2.552 | **0.003** |
| Right superior colliculi | 166.7^a^ | 167.6^a^ | 153.8^b^ | 3.315 | **0.010** |
| Substantia nigra | 18.5^a^ | 18.3^a^ | 16.8^b^ | 0.475 | **0.028** |
| Thalamus | 1334.5^a^ | 1339.6^a^ | 1234.8^b^ | 28.16 | **0.021** |
| ^1^Data presented are least squares means and *P*-values from mixed model 1-way ANOVA.  ^ab^Means lacking a common superscript letter within a row differ (*P < 0.05*).  Abbreviations: DSS, dextrin sodium sulfate; TBCD, cyclodextrin-encapsulated tributyrin; ROI, region of interest; SEM, standard error of the mean. | | | | | |

| **Supplemental Table 2.** Effects of orally supplemented TBCD and DSS-induced colitis on myelin water fraction values of young pigs ^1^ | | | | | |
| --- | --- | --- | --- | --- | --- |
|  | **Treatment** | | | **Pooled SEM** | ***P-Value*** |
| **ROI** | **Control** | **DSS** | **DSS+TBCD** |  |  |
| *n* | 7 | 7 | 7 | - | - |
| Whole Brain | 0.080^a^ | 0.078^b^ | 0.076^b^ | 0.001 | **0.003** |
| Cerebellum | 0.077 | 0.076 | 0.075 | 0.002 | 0.673 |
| Cerebral aqueduct | 0.063 | 0.055 | 0.058 | 0.005 | 0.471 |
| Corpus callosum | 0.068 | 0.062 | 0.070 | 0.003 | 0.255 |
| Fourth ventricle | 0.035 | 0.040 | 0.041 | 0.004 | 0.314 |
| Hypothalamus | 0.079 | 0.078 | 0.079 | 0.002 | 0.759 |
| Lateral ventricle | 0.060 | 0.061 | 0.058 | 0.002 | 0.488 |
| Left caudate | 0.072 | 0.073 | 0.067 | 0.004 | 0.284 |
| Left cortex | 0.081^a^ | 0.078^b^ | 0.076^b^ | 0.001 | **0.001** |
| Left hippocampus | 0.076 | 0.073 | 0.073 | 0.002 | 0.313 |
| Left inferior colliculi | 0.069 | 0.069 | 0.065 | 0.002 | 0.589 |
| Left internal capsule | 0.120 | 0.121 | 0.116 | 0.002 | 0.291 |
| Left olfactory bulb | 0.054 | 0.053 | 0.054 | 0.002 | 0.920 |
| Left putamen-globus pallidus | 0.097 | 0.098 | 0.090 | 0.003 | 0.222 |
| Left superior colliculi | 0.076 | 0.077 | 0.074 | 0.003 | 0.575 |
| Medulla | 0.067 | 0.068 | 0.070 | 0.001 | 0.422 |
| Midbrain | 0.082 | 0.082 | 0.080 | 0.001 | 0.700 |
| Nucleus Accumbens | 0.075 | 0.072 | 0.074 | 0.003 | 0.725 |
| Pons | 0.891 | 0.093 | 0.091 | 0.002 | 0.198 |
| Putamen | 0.096 | 0.096 | 0.092 | 0.002 | 0.403 |
| Right caudate | 0.074 | 0.075 | 0.071 | 0.002 | 0.230 |
| Right cortex | 0.082^a^ | 0.079^b^ | 0.076^c^ | 0.001 | **0.001** |
| Right hippocampus | 0.076 | 0.075 | 0.072 | 0.001 | 0.121 |
| Right inferior colliculi | 0.062 | 0.066 | 0.066 | 0.003 | 0.525 |
| Right internal capsule | 0.118 | 0.126 | 0.116 | 0.003 | 0.083 |
| Right olfactory bulb | 0.054 | 0.053 | 0.053 | 0.002 | 0.966 |
| Right putamen-globus pallidus | 0.095 | 0.094 | 0.094 | 0.002 | 0.992 |
| Right superior colliculi | 0.074 | 0.078 | 0.079 | 0.003 | 0.505 |
| Substantia Nigra | 0.071 | 0.069 | 0.066 | 0.006 | 0.600 |
| Thalamus | 0.092 | 0.092 | 0.090 | 0.002 | 0.733 |
| ^1^Data presented are least squares means and *P*-values from mixed model 1-way ANOVA.  ^a-c^Means lacking a common superscript letter within a row differ (*P < 0.05*).  Abbreviations: DSS, dextrin sodium sulfate; TBCD, cyclodextrin-encapsulated tributyrin; ROI, region of interest; SEM, standard error of the mean. | | | | | |
